# Adverse effects of delayed antimicrobial treatment and surgical source control in adults with sepsis: results of a planned secondary analysis of a cluster-randomized controlled trial

**DOI:** 10.1186/s13054-022-03901-9

**Published:** 2022-02-28

**Authors:** Hendrik Rüddel, Daniel O. Thomas-Rüddel, Konrad Reinhart, Friedhelm Bach, Herwig Gerlach, Matthias Lindner, John C. Marshall, Philipp Simon, Manfred Weiss, Frank Bloos, Daniel Schwarzkopf, the MEDUSA study group, Gernot Marx, Achim Schindler, Tobias Schürholz, Heike Schlegel‑Höfner, Gunther Lehmann, Annett Sander, Steffen Friese, Christian Scholz, Pia Fischer, Christina Fuchs, Lutz Becher, Norbert Salewsky, Torsten Schreiber, Anton Goldmann, Didier Keh, Katrin Schmid, Winfried Menning, Renate Steuckart, Robert Barz, Karin Dey, Meike Fahrenholz, Martin Müller, Herwig Gerlach, Susanne Toussaint, Jörg Brederlau, Friedhelm Bach, Dirk Buschmann, Ingo Gummelt, J. Hoeschen, Marion Klaproth, Ina Vedder, Ulrike Bachmann‑Holdau, Jürgen Eiche, Rolf Hauschild, Martina Lange, Davia Herrmann‑Karbaum, Annette Lubasch, Marcus Rücker, Christian Icke, Alexander Lucht, Andreas Meier‑Hellmann, Jan Wagner, Olaf Arnold, Steffen Kästner, Tobias Clausen, Michael Sternkopf, Robert Voswinckel, T. Benndorf, Christel Eiserloh, Gerhard Kuhnle, Mathias Koch, Manuela Gerber, Matthias Gründling, Liane Guderian, Sven‑Olaf Kuhn, Christian Scheer, Gerd Scheiber, Claudia Matthäus‑Krämer, Bernhard Poidinger, Stefanie D’Aria, Thees Lemke, Birgit Michaelsen, Dirk Schädler, Nina Schulz‑Ruhtenberg, Norbert Weiler, Martin Anetseder, Zoran Textor, Udo Kaisers, Philipp Simon, Matthias Löbe, Frank Meineke, Christine Pausch, Christoph Engel, Georg Braun, Nicole Jensen, Werner Gegenfurtner, Alexander Meinhardt, Robert Schmitt, Andrea Teichert, Klaus‑Dieter Becker, Anja Diers, Florian Jelschen, Andreas Weyland, Frieder Knebel, Thomas Kupfer, Rüdinger Sinz, Petra Bautz, Annemarie Fischer, Armin Seibel, Christoph Fleischhacker, Helene Häberle, Philipp Henn, Friederike Mezger, Peter Rosenberger, Reimer Riessen, Silvia Ziegler, Eberhard Barth, Hendrik Bracht, I. Heymann, A. Hinder, R. Sens, Manfred Weiss, Christof Lascho, Henriette Micke, Falk Schmidt, Stefanie Schilling, Gabriele Wöbker

**Affiliations:** 1 Integrated Research and Treatment Center - Center for Sepsis Control and Care (CSCC), Jena University Hospital, Am Klinikum 1, 07747 Jena, Germany; 2 Department of Anaesthesiology and Intensive Care Medicine, Jena University Hospital, Am Klinikum 1, 07747 Jena, Germany; 3 Department of Anaesthesiology and Operative Intensive Care Medicine (CCM, CVK), Charité Universitätsmedizin Berlin, Corporate Member of Freie Universität Berlin, Humboldt-Universität Zu Berlin, Augustenburger Platz 1, 13353 Berlin, Germany; 4 Berlin Institute of Health, Campus Virchow-Klinikum, Anna-Louisa-Karsch-Straße 2, 10178 Berlin, Germany; 5 Department for Infectious Diseases, Protestant Hospital of Bethel Foundation University Hospital, University of Bielefeld, Bethesdaweg 10, 33617 Bielefeld, Germany; 6 Department for Anaesthesia, Intensive Care Medicine and Pain Management, Vivantes - Klinikum Neukoelln, Rudower Strasse 48, 12351 Berlin, Germany; 7 Department of Anaesthesiology and Intensive Care Medicine, University Hospital Schleswig-Holstein, Campus Kiel, Arnold-Heller-Straße 3, 24105 Kiel, Germany; 8 Li Ka Shing Knowledge Institute, St. Michael’s Hospital, University of Toronto, 209 Victoria St, Toronto, ON M5B 1T8 Canada; 9 Department of Anaesthesiology and Intensive Care, University of Leipzig Medical Centre, Liebigstraße 20, 04103 Leipzig, Germany; 10 Klinik Für Anästhesiologie Und Intensivmedizin, Universitätsklinikum Ulm, Albert-Einstein-Allee 23, 89081 Ulm, Germany; 11 Center for Infectious Diseases and Infection Control, Jena University Hospital, Am Klinikum 1, 07747 Jena, Germany; 12 Department of Intensive Care Medicine, University Hospital RWTH Aachen, Aachen, Germany; 13 Department of Anesthesiology and Intensive Care Medicine, Ilm‑Kreis‑Kliniken Arnstadt, Arnstadt, Germany; 14 Department of Anesthesiology and Intensive Care Medicine, Helios Hospital Aue, Bad Schlema, Germany; 15 Department of Anaesthesia and Intensive Care Medicine, Zentralklinik Bad Berka GmbH, Bad Berka, Germany; 16 Department of Anesthesiology and Operative Intensive Care Medicine, Charité Berlin, Berlin, Germany; 17 Department of Anesthesiology and Intensive Care Medicine, Hufeland‑Klinikum Bad Langensalza, Bad Langensalza, Germany; 18 Department of Anesthesiology and Intensive Care Medicine, Bundeswehrkrankenhaus Berlin, Berlin, Germany; 19 Department of Anesthesiology, Surgical Intensive Care Medicine, and Pain Therapy, Vivantes Klinikum Neukölln Berlin, Berlin, Germany; 20 Department of Intensive Care Medicine, Helios Hospital Berlin‑Buch, Berlin, Germany; 21 Department of Anesthesiology, Emergency and Intensive Care Medicine, and Pain Therapy, Ev. Hospital Bielefeld, Bielefeld, Germany; 22 Department of Anesthesiology, HELIOS‑Hospital St. Josefs‑Hospital Bochum‑Linden, Bochum, Germany; 23 Department of Anesthesiology and Intensive Care Medicine, St. Georg Hospital Eisenach, Eisenach, Germany; 24 Eisenberg. Department of Anesthesiology and Intensive Care Medicine, Hospital Rudolf Elle, Thuringen, Germany; 25 Department of Interdisciplinary Intensive Care and Emergency Medicine, Helios‑Hospital Emil‑von Behring, Berlin, Germany; 26 Department of Anesthesiology and Intensive Care Medicine, Helios‑Hospital Erfurt, Erfurt, Germany; 27 Department of Anesthesiology and Intensive Care Medicine, Catholic Hospital St. Johann Nepomuk Erfurt, Erfurt, Germany; 28 Department of Internal Medicine, Hospital Friedberg, Friedberg, Germany; 29 Department of Anesthesiology and Intensive Care Medicine, SRH Waldklinikum Gera, Gera, Germany; 30 Department of Anesthesiology and Intensive Care Medicine, University Hospital Greifswald, Greifswald, Germany; 31 Department of Anesthesiology and Intensive Care Medicine, Hospital Ilmenau, Ilmenau, Germany; 32 Center for Sepsis Control & Care/Department of Anesthesiology and Intensive Care Medicine, Jena University Hospital, Jena, Germany; 33 Department of Anesthesiology and Intensive Care Medicine, University Hospital Kiel, Kiel, Germany; 34 Department of Anesthesiology and Surgical Intensive Care Medicine, Hospital Landshut‑Achdorf, Landshut, Germany; 35 Department of Anesthesiology and Intensive Care Medicine, University Hospital Leipzig, Leipzig, Germany; 36 Institute of Medical Informatics, Statistics and Epidemiology, University of Leipzig, Leipzig, Germany; 37 Department of Intensive Care and Emergency Medicine, Hospital Meiningen, Meiningen, Germany; 38 Department of Anesthesiology and Intensive Care Medicine, Saale‑Unstrut‑Hospital Naumburg, Naumburg, Germany; 39 Department of Anesthesiology, Intensive Care Medicine, Emergency Medicine, and Pain Therapy, Hospital Oldenburg, Oldenburg, Germany; 40 Department of Anesthesiology and Intensive Care Medicine, Thüringen‑Klinik Pößneck, Pößneck, Germany; 41 Department of Intensive Care and Emergency Medicine, Asklepios Hospital Radeberg, Radeberg, Germany; 42 Department of Anesthesiology, Intensive Care Medicine, and Pain Therapy, Thüringen‑Kliniken Saalfeld, Saalfeld, Germany; 43 Department of Anesthesiology, Intensive Care, and Emergency Medicine, Ev. Jung‑Stilling Hospital Siegen, Siegen, Germany; 44 Department of Anesthesiology, University Hospital Tübingen, Tübingen, Germany; 45 Department of Internal Medicine, University Hospital Tübingen, Tübingen, Germany; 46 University Hospital Medical School Ulm. Clinic of Anaesthesiology, Ulm, Germany; 47 Department of Anesthesiology and Intensive Care Medicine, Hufeland Hospital Weimar, Weimar, Germany; 48 Department of Intensive Care Medicine, Helios Hospital Wuppertal, Wuppertal, Germany

**Keywords:** Sepsis, Infection control, Anti-biotic agents, Time-to-treatment, Patient care bundles, Surgical source control

## Abstract

**Background:**

Timely antimicrobial treatment and source control are strongly recommended by sepsis guidelines, however, their impact on clinical outcomes is uncertain.

**Methods:**

We performed a planned secondary analysis of a cluster-randomized trial conducted from July 2011 to May 2015 including forty German hospitals. All adult patients with sepsis treated in the participating ICUs were included. Primary exposures were timing of antimicrobial therapy and delay of surgical source control during the first 48 h after sepsis onset. Primary endpoint was 28-day mortality. Mixed models were used to investigate the effects of timing while adjusting for confounders. The linearity of the effect was investigated by fractional polynomials and by categorizing of timing.

**Results:**

Analyses were based on 4792 patients receiving antimicrobial treatment and 1595 patients undergoing surgical source control. Fractional polynomial analysis identified a linear effect of timing of antimicrobials on 28-day mortality, which increased by 0.42% per hour delay (OR with 95% CI 1.019 [1.01, 1.028], *p* ≤ 0.001). This effect was significant in patients with and without shock (OR = 1.018 [1.008, 1.029] and 1.026 [1.01, 1.043], respectively). Using a categorized timing variable, there were no significant differences comparing treatment within 1 h versus 1–3 h, or 1 h versus 3–6 h. Delays of more than 6 h significantly increased mortality (OR = 1.41 [1.17, 1.69]). Delay in antimicrobials also increased risk of progression from severe sepsis to septic shock (OR per hour: 1.051 [1.022, 1.081], *p* ≤ 0.001). Time to surgical source control was significantly associated with decreased odds of successful source control (OR = 0.982 [0.971, 0.994], *p* = 0.003) and increased odds of death (OR = 1.011 [1.001, 1.021]; *p* = 0.03) in unadjusted analysis, but not when adjusted for confounders (OR = 0.991 [0.978, 1.005] and OR = 1.008 [0.997, 1.02], respectively). Only, among patients with septic shock delay of source control was significantly related to risk-of death (adjusted OR = 1.013 [1.001, 1.026], *p* = 0.04).

**Conclusions:**

Our findings suggest that management of sepsis is time critical both for antimicrobial therapy and source control. Also patients, who are not yet in septic shock, profit from early anti-infective treatment since it can prevent further deterioration. *Trial registration* ClinicalTrials.gov (NCT01187134). Registered 23 August 2010, NCT01187134

**Supplementary Information:**

The online version contains supplementary material available at 10.1186/s13054-022-03901-9.

## Background

Sepsis is a life-threatening organ dysfunction caused by a dysregulated host response to infection [1]. International clinical practice guidelines define sepsis as an emergency, demanding treatment with broad spectrum antibiotics [2]. They recommend antimicrobial treatment within 1 h in patients with septic shock or a high likelihood of sepsis, and treatment within 3 h in patients with possible sepsis without shock and persisting concerns of infection [2]. Evidence for the effect of timing of antimicrobial therapy on mortality is contradictive [3–6].

It is argued that prompt antimicrobials within the first hours might be warranted for the most severely ill patients with septic shock, only.[5, 7, 8]. Therefore, current guidelines reduced the evidence rating for immediate treatment from moderate to low in septic shock and from moderate to very low in sepsis without shock [2, 9]. However, current evidence is based on systematic reviews that comprised numerous studies with several acknowledged methodological flaws [3, 4, 6]. These include usage of routine databases without prospective inclusion of patients with sepsis, non-physiologic definition of time zero—e.g. by emergency triage time or first sepsis-related treatment, not adequately adjusting for confounders, and not investigating treatment effects separately for patients without and with septic shock [5, 8].

Guidelines also advice as best practice to conduct surgical source control as soon as medically and logistically possible [2]. However, this best practice statement is based on few studies only, mostly conducted among samples of single specific disease entities—like necrotizing fasciitis—and with diverging results [10–14].

To overcome these limitations, we analysed a large data set from the prospective cluster-randomized quality improvement MEDUSA trial to assess the impact of timing of antimicrobial therapy and source control on mortality.

## Methods

### Study design and setting

The aim of this observational cohort study was to investigate the effect of timing of anti-infective treatment on outcomes in patients with severe sepsis. Primary outcome was 28-day mortality. Secondary outcomes were hospital length-of-stay of survivors, progression from severe sepsis to septic shock, and success of surgical source control. Primary exposures were timing of antimicrobial therapy and timing of surgical source control. This study was conducted as a planned secondary analysis using data from the two-arm cluster-randomized controlled MEDUSA trial (Medical Education for Sepsis Source Control and Antibiotics, ClinicalTrials.gov Identifier NCT01187134) [15].

The MEDUSA trial aimed to improve anti-infective measures for patients with sepsis. Hospitals were randomized to either an intervention group receiving a multifaceted educational intervention, or a control group receiving only standard lectures on sepsis treatment. Results from this trial, which has been conducted from July 2011 to May 2013, have been reported previously [15]. The sample of the now reported secondary analysis additionally includes data gathered during a second intervention phase conducted from September 2013 to May 2015, which implemented an enhanced version of the educational intervention among former control hospitals. Intervention group and study phase are controlled as a possible confounder in the current observational secondary analysis, but the evaluation of the enhanced intervention itself will be reported in a separate article. A convenience sample of 40 German hospitals involved in primary sepsis care was included. Patients with severe sepsis were prospectively included by intensive care physicians on the participating intensive care units. All sepsis diagnoses were reevaluated on day 5 after screening; only cases with confirmed diagnoses were included to the study. Data were abstracted from the medical records and documented using the data management software OpenClinica® (OpenClinica, LLC, Waltham, MA, USA). This study was performed in line with the principles of the Declaration of Helsinki and approved by the local ethics committees responsible for a participating institution. The need for informed consent was waived since the interventions comprised quality improvement measures.

### Patient sample

All consecutive adult patients treated in the ICU for proven or suspected infection with at least one new organ dysfunction related to the infection were eligible for inclusion. Organ dysfunctions were defined as follows: acute encephalopathy, thrombocytopenia defined as a platelet count < 100,000/ μl or a drop in platelet count > 30% within 24 h, arterial oxygen partial pressure < 10 kPa (75 mmHg) when breathing room air or partial pressure of arterial oxygen/fraction of inspired oxygen ratio < 33 kPa (< 250 mmHg), renal dysfunction defined as oliguria (diuresis ≤ 0.5 ml/kg body weight/hour) despite adequate fluid resuscitation or an increase of serum creatinine more than twice the local reference value, metabolic acidosis with a base excess <  − 5 mmol/l or a serum lactate > 1.5 times the local reference value, and arterial hypotension defined as systolic arterial blood pressure < 90 mmHg or mean arterial blood pressure < 70 mmHg for > 1 h despite adequate fluid loading or vasopressor therapy at any dosage to maintain higher blood pressures [16]. SIRS-criteria did not need to be present to define sepsis. Patients were excluded, if there was a therapy limitation at the onset of sepsis, if they were not treated in the participating ICU after sepsis diagnosis, or if sepsis therapy had already been started in another hospital.

Preliminary findings on the effects of timing of antimicrobials and source control on mortality were presented in a previous publication [15]. These preliminary analyses were conducted based on cases included during the first intervention phase of the trial. The now reported detailed analyses have not been conducted before and are based on additional patients included in the second intervention phase. They surpass previous analyses by excluding patients receiving anti-infective treatment before first organ dysfunction to prevent bias, excluding indicators of illness severity assessed after sepsis onset from the set of considered confounders, investigating the linearity of the effect of timing by fractional polynomials, investigating the effect separately in cases with and without shock as well as other subgroups, and additionally to mortality investigating the effect on progression to shock, success of source control, and hospital length-of-stay.

### Outcomes and exposures

The primary outcome was 28-day mortality. Secondary outcomes were hospital length-of-stay for survivors, success of source control, and progression to septic shock. Success of source control was assessed by the treating physicians and to their own discretion; blinding to outcome was not intended by the study protocol. Septic shock was defined by the need to administer vasoactive agents to maintain mean arterial pressure ≥ 70 mmHg. Presence of shock was assessed during two timeframes: during the first 12 h after sepsis onset, and between 12 and 24 h after sepsis onset. Therefore, we defined progression to septic shock as new onset of septic shock between 12 and 24 h among those patients not in shock during the first 12 h.

Onset of sepsis was defined as the earliest time of the first infection-related organ dysfunction as documented in the patient’s medical record. All available information was assessed to identify time zero, including patient charts from the general ward or emergency department. Primary exposures were timing of antimicrobial therapy and timing of surgical source control. Timing was used as continuous variable, however excluding cases with times < 0 h and times > 48 h. Cases with times < 0 h were excluded from the respective analysis, since these cases would represent a very specific subsample: patients, who develop infection related organ dysfunction and the need of critical care despite anti-infective treatment before sepsis onset. Cases with timing > 48 h were excluded as outliers. Timing of antimicrobials was measured as first administration. Timing of source control was defined by the first cut. We also investigated the effect of timing of surgical source control on success of surgical source control, and of timing to antimicrobial treatment and timing of surgical source control on the risk of progression to septic shock, as well as the effects of success of surgical source control on mortality and length-of-stay.

### Statistical analysis

Baseline characteristics of patients were statistically described stratified by timing of antimicrobial therapy and timing of surgical source control. Logistic regressions were used to analyse effects of exposures on mortality, success of source control, and progression to septic shock. Effects on hospital length-of-stay were analysed using linear regression after log-transformation of length-of-stay. Since it was suggested that the effect of timing of treatment on mortality might be non-linear [5], we used two strategies to investigate the linearity of the effect: first, timing was analysed as a categorical variable including start of treatment within one hour, one to three hours, three to six hours, or after more than six hours to allow comparison with previous research [17]; second, we used fractional polynomials which operationalize the functional form of an effect and test for deviations from linearity [18].

All regression analyses adjusted for covariates. Covariates for risk-adjustment were chosen a priori as potential confounders of the effects between timing of treatment and outcomes based on clinical experience and previous studies [15, 19–21]. These variables included age and gender, origin of infection, location of the patient at the onset of sepsis, focus of infection, microbiological confirmation of infection, study phase (trial vs. surveillance phase), and the group the hospital was randomized to (intervention vs. control during the trial phase). To measure severity of critical illness after sepsis onset, vasopressor use within the first 12 h, as well as the maximum values within the first 24 h after sepsis onset of SAPS-II, lactate in mmol/l, platelets, and base excess were used [22, 23]. Two risk-adjustment models were developed: In the primary analyses covariates on the severity of critical illness during the first 24 h were not included, since these might themselves be influenced by timing to treatment, as well as in response to recent arguments that effects of timing of antimicrobial therapy might only be present after statistical adjustment for severity of illness [24]. Covariates on the severity of critical illness were then included in the second risk model to conduct sensitivity analyses. Both resulting models showed adequate calibration (Hosmer–Lemeshow goodness-of-fit test: *p* = 0.077 and *p* = 0.219, Additional File 1: SFig. 1); including covariates on the severity of critical illness increased discrimination and explained variance (area under the receiver operating characteristic: 0.68 vs. 0.76; *R*^*2*^: 0.08 vs. 0.18). STables 1 and 2 in Additional File 2 present the effects of individual risk-factors. To control for clustering, regression analyses were done by mixed models with a random intercept.

To analyse the combined effects of delay to antimicrobial therapy and delay to surgical source control on mortality, both were included in one logistic regression and an interaction effect between the two was tested.

#### Subgroup and sensitivity analyses

To test, if the effect of timing of treatment on mortality was different comparing subgroups, interaction terms between timing and pre-specified effect modifiers were introduced into the regression models. Possible modifiers were chosen based on previous research [19]: Vasopressor use within the first 12 h after onset of sepsis (yes vs. no), origin of infection (community acquired, nosocomial on ICU or intermediate care unit, nosocomial on general ward), location of the patient at onset of sepsis (ICU or intermediate care unit, ambulance service or emergency department, general ward, operating room), microbiological confirmation of infection (yes vs. no), bacteraemia and type of identified pathogen (gram positive, gram negative, other or several pathogens, no pathogen detected, no blood culture taken), change of antimicrobial therapy within 5 days (escalation, de-escalation, no change), surgical source control conducted (yes vs. no; in analyses on timing of antimicrobials), or success of surgical source control (yes vs. no; in analysis on timing of surgical source control), respectively.

As a sensitivity analysis, the effects of timing of treatment on mortality were assessed while also adjusting for covariates measuring the severity of critical illness within the first 24 h after onset of sepsis.

All analyses were conducted at a significance level of *α* < 0.05 using the statistical software *R*, version 4.1.2 (R Foundation for Statistical Computing, Vienna, Austria). The amount of missing values on exposures, outcomes, and confounders for the primary analyses and subgroup analyses was roughly ≤ 5%. Therefore, analysis of complete data was regarded as suitable strategy for treating missing data [25].

## Results

### Characteristics of patients and time to treatment

Characteristics of participating hospitals are presented in Additional File 2: STable 3. Overall, 6576 patients with severe sepsis or septic shock were included, of whom 6514 received antimicrobial treatment for the infection that caused sepsis; antibiotics were started after the first organ dysfunction and within 48 h in 4792 patients, of which 1687 (35%) had been included in the second intervention phase of the trial and not been analysed before (see Additional File 1: SFig. 2 for study flow chart and Additional File 2: STable 4 for characteristics of excluded patients). These patients showed a mortality of 32%. Median age was 70 years, 62% were male, 47% had a community acquired infection; respiratory infections were the most common focus (43%), 75% received vasopressors during the first 12 h after sepsis onset (Table 1). Surgical source control was undertaken among 2562 patients, 1595 of whom received it after the first organ dysfunction and within 48 h (see Additional File 1: SFig. 2 for study flow chart and Additional File 2: STable 5 for characteristics of excluded patients). These patients showed a mortality of 33%. The most common focus was abdominal infection (73%), and 85% received vasopressors during the first 12 h (Table 2).

**Table 1:** Baseline characteristics of patients stratified by timing of start of antimicrobial treatment

Variable	No. of patients with complete data	All patients (*N* = 4792)	Timing of antimicrobial therapy		
			Within 1 h (*N* = 1311)	More than 1 h (*N* = 3481)	*p* value
Time to beginning of antimicrobial therapy (minutes)	4792	150 [60, 378.5]	30 [15, 50]	240 [120, 518]	–
Age	4791	70 [59, 77]	71 [60, 77]	70 [59, 77]	0.435
Sex: male	4792	2986 (62.3%)	800 (61%)	2186 (62.8%)	0.27
Origin of infection: Community acquired	4791	2273 (47.4%)	779 (59.4%)	1494 (42.9%)	≤ 0.001
Nosocomial (ICU/IMC)		1112 (23.2%)	208 (15.9%)	904 (26%)	
Nosocomial (general ward)		1406 (29.3%)	324 (24.7%)	1082 (31.1%)	
Location at onset of sepsis: ICU	4792	2233 (46.6%)	559 (42.6%)	1674 (48.1%)	≤ 0.001
Emergency department		948 (19.8%)	333 (25.4%)	615 (17.7%)	
Operating room		428 (8.9%)	167 (12.7%)	261 (7.5%)	
General ward		692 (14.4%)	127 (9.7%)	565 (16.2%)	
Ambulance service		211 (4.4%)	48 (3.7%)	163 (4.7%)	
IMC		280 (5.8%)	77 (5.9%)	203 (5.8%)	
Focus of infection: respiratory	4789	2057 (43%)	510 (38.9%)	1547 (44.5%)	≤ 0.001
Focus of infection: abdominal	4789	1657 (34.6%)	464 (35.4%)	1193 (34.3%)	0.474
Focus of infection: urogenital	4789	695 (14.5%)	209 (16%)	486 (14%)	0.089
Focus of infection: bones/soft tissue/wound	4789	531 (11.1%)	154 (11.8%)	377 (10.8%)	0.38
Focus of infection: other/unknown	4789	644 (13.4%)	161 (12.3%)	483 (13.9%)	0.154
Infection microbiologically confirmed	4781	3514 (73.5%)	965 (73.7%)	2549 (73.4%)	0.854
Bacteremia: Gram positive	4754	806 (17%)	223 (17.2%)	583 (16.9%)	0.005
Gram negative		712 (15%)	226 (17.4%)	486 (14.1%)	
Other/several		166 (3.5%)	41 (3.2%)	125 (3.6%)	
No pathogen detected		2281 (48%)	627 (48.3%)	1654 (47.9%)	
No blood culture taken		789 (16.6%)	182 (14%)	607 (17.6%)	
Vasopressor use within 12 h after first organ dysfunction	4781	3595 (75.2%)	982 (75.2%)	2613 (75.2%)	1

Descriptive statistics given as N (%) or median [interquartile range]. *p* values obtained by Mann-Whitney-U test, Fisher’s exact test or Pearson’s Chi-squared test, as appropriate

ICU: intensive care unit; IMC: intermediate care unit

**Table 2:** Baseline characteristics of patients stratified by timing of surgical source control

Variable	No. of patients with complete data	All patients (*N* = 1595)	Timing of surgical source control		
			Whithin 6 h (*N* = 924)	More than 6 h (*N* = 671)	*p* value
Time to surgical source control (minutes)	1595	275 [81, 810]	109 [38, 225]	950 [593, 1500]	–
Age	1595	70 [59, 77]	70 [60, 78]	69 [59, 76]	0.152
Sex: male	1595	934 (58.6%)	538 (58.2%)	396 (59%)	0.758
Origin of infection: Community acquired	1595	795 (49.8%)	507 (54.9%)	288 (42.9%)	≤ 0.001
Nosocomial (ICU/IMC)		292 (18.3%)	111 (12%)	181 (27%)	
Nosocomial (general ward)		508 (31.8%)	306 (33.1%)	202 (30.1%)	
Location at onset of sepsis: ICU	1595	573 (35.9%)	255 (27.6%)	318 (47.4%)	≤ 0.001
Emergency department		298 (18.7%)	170 (18.4%)	128 (19.1%)	
Operating room		344 (21.6%)	324 (35.1%)	20 (3%)	
General ward		232 (14.5%)	107 (11.6%)	125 (18.6%)	
Ambulance service		39 (2.4%)	22 (2.4%)	17 (2.5%)	
IMC		109 (6.8%)	46 (5%)	63 (9.4%)	
Focus of infection: respiratory	1595	171 (10.7%)	69 (7.5%)	102 (15.2%)	≤ 0.001
Focus of infection: abdominal	1595	1161 (72.8%)	705 (76.3%)	456 (68%)	≤ 0.001
Focus of infection: urogenital	1595	121 (7.6%)	60 (6.5%)	61 (9.1%)	0.056
Focus of infection: bones/soft tissue/wound	1595	272 (17.1%)	135 (14.6%)	137 (20.4%)	0.003
Focus of infection: other/unknown	1595	117 (7.3%)	59 (6.4%)	58 (8.6%)	0.098
Infection microbiologically confirmed	1592	1239 (77.8%)	696 (75.6%)	543 (80.9%)	0.012
Bacteremia: gram positive	1587	224 (14.1%)	99 (10.8%)	125 (18.7%)	≤ 0.001
Gram negative		206 (13%)	117 (12.7%)	89 (13.3%)	
Other/several		54 (3.4%)	29 (3.2%)	25 (3.7%)	
No pathogen detected		720 (45.4%)	421 (45.9%)	299 (44.7%)	
No blood culture taken		383 (24.1%)	252 (27.5%)	131 (19.6%)	
Vasopressor use within 12 h after first organ dysfunction	1590	1346 (84.7%)	830 (90.2%)	516 (77%)	≤ 0.001

Descriptive statistics given as *N* (%) or median [interquartile range]. *p*-values obtained by Mann–Whitney-U test, Fisher’s exact test or Pearson’s Chi-squared test as appropriate

ICU: intensive care unit; IMC: intermediate care unit

Median delay until antimicrobial therapy was 150 min (1st quartlile: 60, 3rd quartile: 378.5), 3481 (73%) of patients received antimicrobials after more than 1 h. Median delay to surgical source control was 275 min (1st quartile: 81, 3rd quartile: 810), 671 (42%) patients underwent source control after more than 6 h (Additional File 1: SFig. 3 a-d). Baseline characteristics of patients stratified by timing of antimicrobial therapy and surgical source control are presented in Tables 1 and 2, respectively. Patients who received antimicrobial therapy within the first hour and patients who received surgical source control within the first 6 h more often had a community acquired infection when compared to patients with delayed therapy.

### Effects of time to treatment on mortality

**Fig. 1:**
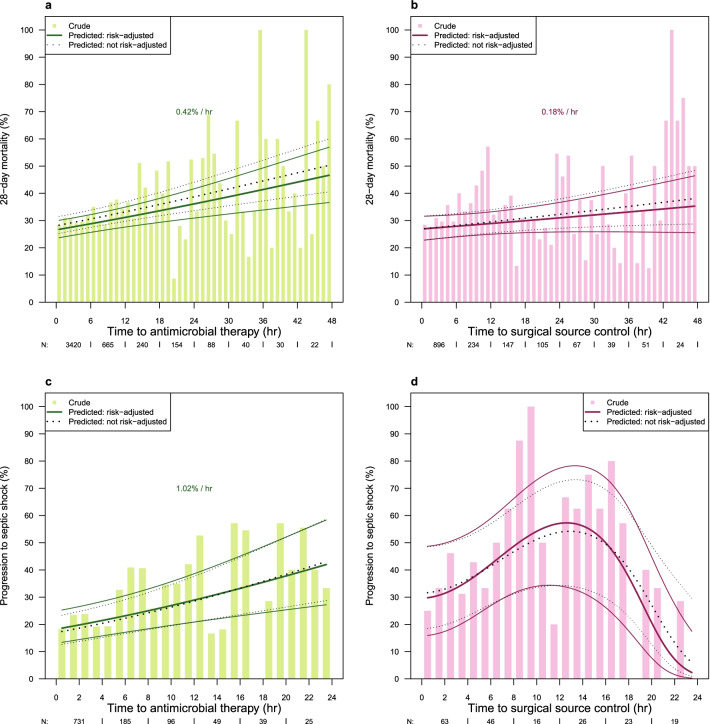
Crude outcomes and predicted outcomes depending on time to treatment. Presented are the risk (bars) and predicted risk (lines) across a range of time after onset of sepsis. Bold lines present the predicted risk with 95% confidence interval for a typical patient from a model adjusting for covariates, dotted lines present the predicted risk with 95% confidence interval from a model without adjusting for covariates. The effect of timing was tested for linearity by fractional polynomials at significance level 0.05; in panels **a** to **c** timing is treated as linear, since no significant deviation from linearity was found. Models adjusted for the following covariates: age and gender, origin of infection, location of the patient at the onset of sepsis, focus of infection, microbiological confirmation of infection, study phase (trial vs. surveillance phase), and group the hospital was randomized to (intervention vs. control). **a** Effect of time to antimicrobial therapy within the first 48 h on 28-day mortality. *N* = 4792 cases were treated with antimicrobials within 48 h after sepsis onset, of which 4659 (97%) cases had complete data on outcome and covariates for analysis. **b** Effect of timing of surgical source control within the first 48 h on 28-day mortality. *N* = 1595 cases had surgical source control within 48 h after onset of sepsis, of which 1563 (98%) cases had complete data on outcome and covariates for analysis. **c** Effect of time to antimicrobial therapy on the risk of progression to septic shock between 12 and 24 h after onset of sepsis. *N* = 1129 received their first antimicrobial treatment within 24 h after sepsis onset and were at risk of developing shock between 12 and 24 h, of these 1125 (99%) cases had complete data on outcome and covariates for analysis. **d** Effect of time to surgical source control on the risk of progression to septic shock between 12 and 24 h after onset of sepsis. Effect fitted by the fractional polynomial ${\left(\left(x+0.1\right)/10\right)}^{2}+{\left(\left(x+0.1\right)/10\right)}^{3}$ , where x is the timing of surgical source control. *N* = 193 cases did undergo a surgical source control within 24 h after sepsis onset and were at risk of developing shock between 12 and 24 h, of which 193 cases had complete data on outcome and covariates for analysis

**Fig. 2:**
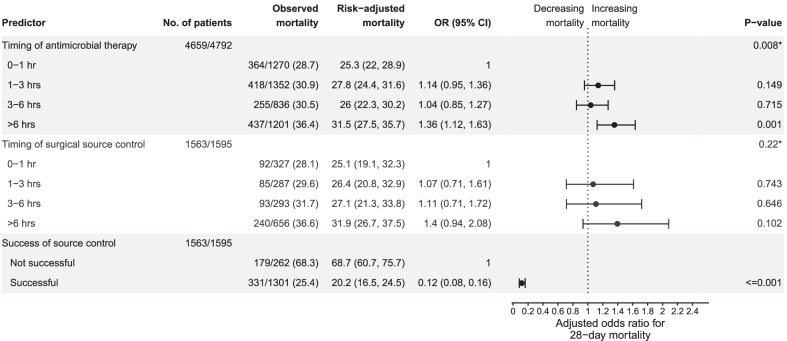
Effects of antimicrobial therapy and of surgical source control on 28-day mortality. Effects were tested in a logistic hierarchical linear model with a random intercept adjusting for covariates. Risk-adjusted mortality estimates were obtained as predictive margins that were calculated for the average of continuous variables and for the most common category of categorical variables. No. of patients gives the number of cases with complete data compared to the total number of patients suitable for the respective analysis. * marks the p-value of the overall test of significance for the categorical variables on timing conducted by a likelihood-ratio test, while the other p-values give the results of tests of single categories against the reference category. Models adjusted for the following covariates: age and gender, origin of infection, location of the patient at the onset of sepsis, focus of infection, microbiological confirmation of infection, study phase (trial vs. surveillance phase), and group the hospital was randomized to (intervention vs. control)

Without risk-adjustment, time between onset of sepsis and start of antimicrobial therapy had a significant effect on 28-day mortality with an unadjusted OR of 1.02 (95% CI 1.012, 1.029; *p* ≤ 0.001). The effect was also significant when adjusted for covariates (adjusted OR per hour with 95% CI 1.019 [1.01, 1.028], *p* ≤ 0.001) and corresponded to an increase of 0.42% per hour delay for a typical patient (Fig. 1 a). Pairwise comparisons of discrete time intervals with antimicrobial treatment within 1 h revealed non-significant effects for timing of 1–3 h (OR = 1.14 [0.95, 1.36]) and 3–6 h (OR = 1.04 [0.85, 1.27]) but a significantly increased odds of hospital mortality for time greater than 6 h (OR = 1.36 [1.12, 1.63], Fig. 2).

Delay of surgical source control showed a significant relation to an increase in mortality by an unadjusted OR of 1.011 (1.001, 1.021; *p* = 0.03), but this relation failed to reach statistical significance when covariates were adjusted (Fig. 1 b, OR = 1.008 [0.997, 1.02], *p* = 0.143). Also pairwise comparisons of discrete time intervals did not show significant effects when adjusted for confounders (overall *p* = 0.22, Fig. 2).

Combined effects of timing of antimicrobial therapy and timing of surgical source control were investigated among 1222 patients, which had received both treatments between sepsis onset and 48 h. There was no significant interaction effect between both (OR of 1 [0.998, 1.001]; *p* = 0.577), meaning that delay in one treatment did not change the effect of a delay in the other treatment, but both acted additively (see Additional File 2: STable 6).

### Effects of time to treatment on success of source control and progression to septic shock

Source control was successful in 1333 of 1595 patients (83.6%). Successful source control was associated with decreased mortality (OR = 0.12 [0.08, 0.16], *p* ≤ 0.001, Fig. 2). A delay of surgical source control was associated with a reduced rate of successful source control when not adjusted for confounders (OR per hour: 0.982 [0.971, 0.994], *p* = 0.003), but the adjusted effect was not significant (OR = 0.991 [0.978, 1.005], *p* = 0.197, Additional File 1: SFig. 4).

A delay in antimicrobial treatment was associated with a higher risk of progression to septic shock between 12 and 24 h after sepsis onset (OR = 1.051 [1.022, 1.081] per hour, *p* ≤ 0.001, based on 1129 patients, of which 291 (25.8%) progressed to shock, Fig. 1c). Unexpectedly, time to surgical source control did not show a linear relation to the risk of progression to septic shock, but a reverse-u-shaped effect, with the risk increasing until 12 h and then decreasing again (*p* = 0.002, based on 193 cases, of which 82 progressed to shock, Fig. 1d).

### Effects of time to treatment on hospital length-of-stay of hospital survivors

Length-of-stay for hospital survivors was longer if the start of antimicrobial therapy or surgical source control was delayed (ratio of geometric means with 95% CI 1.02 [1, 1.03], *p* = 0.023, and 1.04 [1.01, 1.07], *p* = 0.003, respectively, Additional File 2: STable 7). Successful source control was associated with reduced hospital length of stay (0.72 [0.61, 0.86], *p* ≤ 0.001).

### Subgroup analyses

**Fig. 3:**
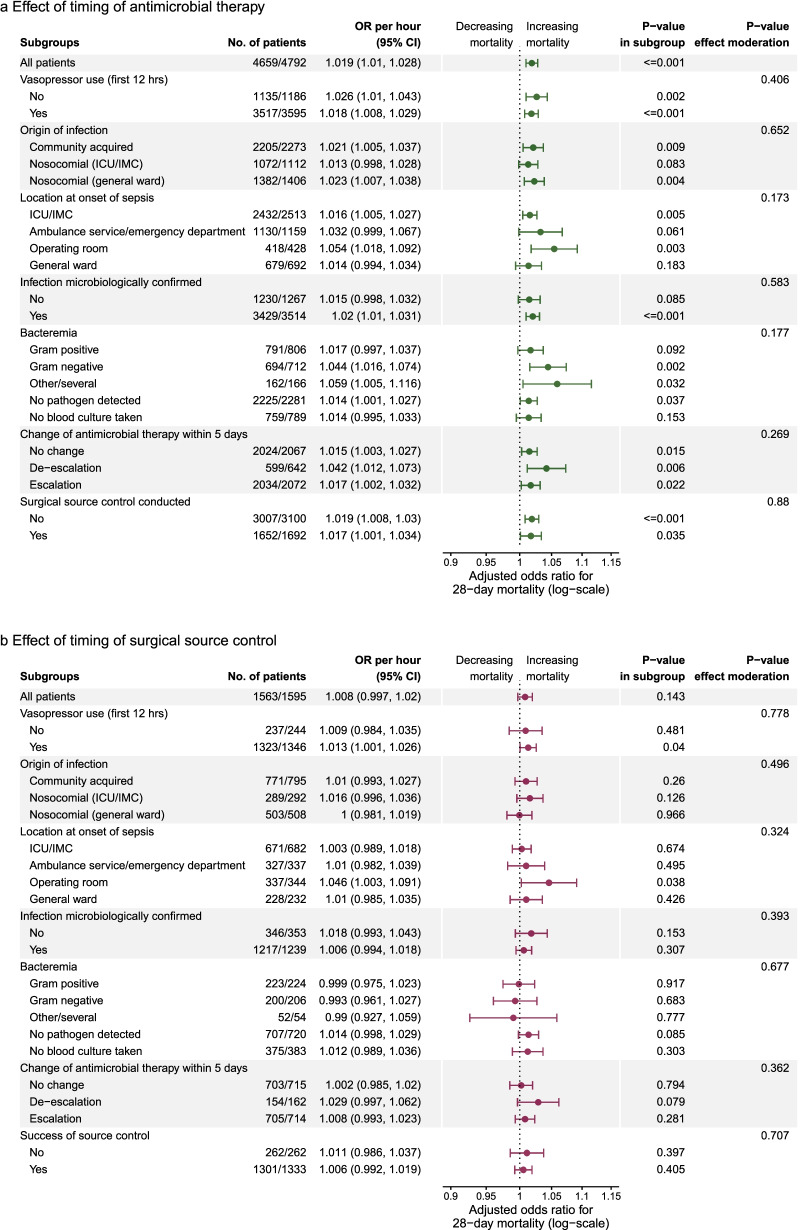
Test of effect moderation of timing of treatment in prespecified subgroups for time from onset of sepsis to start of antimicrobial therapy (panel **a**) and conduction of surgical source control (panel **b**) on 28-day mortality. OR: odds ratio per hour delay of treatment. Only cases with times between 0 and 48 h were considered. Effect moderation was tested by introducing individual interaction terms between respective effect moderator and the effect of timing of antimicrobial therapy or timing of surgical source control, respectively, in the logistic regression models. Regression models were calculated by logistic hierarchical linear models with a random intercept. No. of patients gives the number of cases with complete data compared to the total number of patients within the respective subgroup. Models adjusted for the following covariates: age and gender, origin of infection, location of the patient at the onset of sepsis, focus of infection, microbiological confirmation of infection, study phase (trial vs. surveillance phase), and group the hospital was randomized to (intervention vs. control). ICU: intensive care unit; IMC: intermediate care unit

Delayed antimicrobial treatment was associated with increased odds of death both among patients not receiving vasopressors within the first 12 h (adjusted OR = 1.026 [1.01, 1.043] per hour, *p* = 0.002) and patients receiving vasopressors (adjusted OR = 1.018 [1.008, 1.029], *p* ≤ 0.001; test of difference between odds-ratios: *p* = 0.406). A delay of surgical source control was significantly related to increased mortality only among patients receiving vasopressors within the first 12 h (adjusted OR = 1.013 [1.001, 1.026], *p* = 0.04), but not among patients without shock (adjusted OR = 1.009 [0.984, 1.035], *p* = 0.481, test of difference between odds-ratios: *p* = 0.778). All subgroup analyses are presented in Fig. 3a, b.

### Sensitivity analyses

When covariates on the severity of acute illness during the first 24 h after onset of sepsis were included, the effect of time to antimicrobial therapy remained largely unchanged compared to the primary analysis (Additional File 2: STable 8). Contrary to the primary analysis, time to source control showed a significant effect on 28-day mortality, when these additional covariates were included (OR = 1.02 [1.01, 1.04]; *p* = 0.001).

## Discussion

The key finding of our study is that a delay in time to treatment of infection is associated with an increase in 28-day mortality in patients with sepsis or septic shock. A delay in time to antibiotics increased the risk of death both among patients with and without septic shock and elevated the risk for progression from sepsis to septic shock. The relationship of timing of surgical source control and patient outcome was less consistent. When adjusted for confounders, it was only shown among patients with septic shock.

Previous research shows a large variation in the effect of timing of antimicrobial treatment on mortality in patients with sepsis. Two meta-analyses including studies until 2015 and 2016, respectively, revealed opposite results: Sterling et al. did not find a significant effect of timing on mortality by focussing on observed mortality [3]. Johnston et al. also included risk-adjusted effects and reported a significant decrease in odds of death in patients with early antimicrobial therapy [6]. Recent larger studies showed significant but small effects on mortality between 0.3 and 1.4% increased risk per hour [19, 20, 26–28]. This aligns with our findings of 0.4% increased risk of death per hour delay. This effect was significant both unadjusted and adjusted for possible confounders.

While most previous studies did not separate between sepsis and septic shock [5], our analyses showed a significant and similar effect of timing of antimicrobial treatment on mortality in both subgroups. Previous studies that compared patients with and without shock partly found no effect of delayed antimicrobials at all [29], smaller effects among patients without shock [19, 26], or virtually equal effects among both groups [20, 27]. A recent study assessing all patients with infection admitted via the emergency department found a larger effect of delayed antimicrobials in a higher severity subgroup mostly defined by elevated lactate levels compared to the much larger group with lower severity at presentation [28]. Our data also show that the delay of antimicrobials increases the risk of progression to septic shock, as has also been reported in two other studies [30, 31].

Weinberger et al. proposed a non-linear relation between delay of antimicrobials and mortality, claiming that there would be virtually no increase in mortality if delay was below six hours [5, 7]. This argumentation has also been used to question the 1-h goal demanded by the sepsis guidelines of 2016 [5, 8]. Most previous studies were methodologically inappropriate to investigate this question, since they used emergency department triage as time zero [4, 5, 17], where it is unclear how long sepsis persisted before that point in time. Using the documented occurrence of the first organ dysfunction as time zero allows more valid conclusions. Weinberger et al. suggested to calculate distinct adjusted estimates for the association between each hourly interval until antibiotics to test for a steady increase in mortality [5]. We argue that such pairwise comparisons between short time intervals are prone to be underpowered and biased by random error and unknown confounders. Likewise, we found that there was an increase of risk-adjusted mortality by 2.4% if treatment was delayed for 1 to 3 h, but this difference was not significant. Explicitly testing for non-linearity of the effect of a continuous predictor is more informative than pairwise comparisons between intervals. Fractional polynomials are a method to test for non-linearity and to calculate an appropriate non-linear functional form for an effect in regression analyses [18, 19]. Using this method, we did not find significant evidence for a non-linear effect of delay of antimicrobials on mortality, which results in assuming a linear effect. There will be no conclusive evidence from observational studies, if it is safe or not safe to postpone anti-infective treatment for a short period of time among some patients with suspected sepsis. Responsible clinicians need to weigh up the competing risks of deterioration of a patient with sepsis vs. treating a patient possibly not having an infection with broad spectrum antibiotics. The new sepsis guidelines of 2021 reflect this reasoning by suggesting a time‑limited course of rapid assessment of infectious vs. non-infectious causes among patients with possible sepsis without shock and, if concern for infection persists, the administration of antimicrobials within 3 h [2]. Delays in treatment are usually not based on purposeful clinical decision-making but on lacking awareness, late recognition of clinical deterioration, shortcomings in communication and other organizational barriers to early diagnosis and treatment [38]. Therefore, our results support initiatives to raise awareness for sepsis as a medical emergency.

Studies on delay in surgical source control were mostly conducted among patients with single disease entities such as necrotizing fasciitis, peptic ulcer perforation, cholangitis, infected pancreatic necrosis or urinary tract infections and mostly had small samples with heterogeneous disease severity [2, 10–12, 32–34]. Two recent large studies among patients with sepsis [13] and septic shock [14] did not find a significant increase in mortality by delayed source control. In unadjusted analyses, we found a significant effect of timing of source control on mortality, while adjusted for possible confounders this effect only proved to be significant among patients with septic shock. Success of source control itself is a strong predictor of survival [35, 36]. Our study additionally found a delay in source control to decrease the chances of success, but again only in the unadjusted analyses and not in the analysis adjusted for confounders. Therefore, the sensitivity of effects of timing of treatment on outcome to confounder adjustment, which has been extensively discussed regarding antibiotics [5], also applies to analyses of timing of source control.

Additionally, delays in surgical source control in relation to risk of progression to shock within 12 to 24 h after sepsis onset showed an unexpected reversed U-shaped curve. Such a U-shape may be the result of treatment decisions prior to surgery as physicians may decisively postpone surgery in patients, which might need or might benefit from additional stabilization of hemodynamics and physiologic status prior to surgical source control [37]. Likewise, surgeons might have delayed intervention due to diagnostic uncertainty in patients with vague or subtle symptoms, which might have had better outcomes. Therefore, on the one hand, our results indicate that early surgical source control is warranted in patients with sepsis—especially in patients with shock. On the other hand, they hint at the complexities of the dynamics of symptoms, illness progression, and clinical decision-making that cannot be fully measured or operationalized in an observational study.

This study has several strengths. It is based on a large sample of prospectively included patients with sepsis combining data on both timing of antimicrobial therapy and timing of source control. It is less biased by methodological flaws, since it is one of the few studies using physiological criteria of sepsis onset as time zero, while most previous studies—especially those with large sample sizes—used timing of emergency department triage or beginning of treatment [3–6, 19, 26, 27]. It is also one of the few studies using a standardized follow-up period instead of hospital mortality. Most previous studies also did not adjust adequately for possible confounders [5]. In our study, the effects of delayed antimicrobial therapy were significant both without risk adjustment as well as with adjustment including presenting symptoms and illness severity, suggesting that the effect of timing was not merely an artefact of statistical adjustment [8].

The study also has limitations. Definition of time zero by the review of physiological parameters in patient charts has a problem of imperfect objectivity and reliability due to incomplete measurement or documentation in routine care [39]. This might have biased the effect of delay of treatment found in our study. Since ordering time has not been assessed in our study, we were not able to separate between time of recognition of sepsis and time of antimicrobial treatment [17]. Also, sensitivity of pathogens to the first antibiotics was not systematically assessed and could therefore not be analysed. Little information was available about pre-existing risk-factors such as comorbidities or presenting signs and symptoms like vital signs and laboratory values at onset of sepsis, which are two important gaps to the risk-adjustment [5]. A delay in source control might be due to the need to stabilize patients first, but we had no sufficient data to identify respective patients in our analysis. Our study was conducted among German hospitals, only, but results are comparable to international studies [19, 20, 26, 30]. This study did only include patients with sepsis in need of critical care. Therefore, it was not possible to investigate possibly preventive effects of anti-infective interventions before occurrence of infection related organ dysfunctions. In addition, the findings of this study cannot be applied to less severely ill patients not needing intensive care. Study physicians were not blinded to outcome when evaluating the success of surgical source control, which might have caused a bias of judgement of success.

## Conclusions

Our findings that delays in antimicrobial treatment after onset of sepsis and surgical source control after onset of septic shock were associated with increased 28-day mortality, increased the risk of progression from sepsis to septic shock and decreased probability of successful source control, suggest that for the management of sepsis time matters both for antimicrobial therapy and source control. Also patients, who are not yet in septic shock, profit from early anti-infective treatment since it can prevent further deterioration.

## Supplementary Information

**Additional file 1.** **Supplemental Figures. SFigure 1.** Calibration belt of the risk-adjustment-model for 28-day-mortality. The figure shows observed 28-day mortality (y-axis) compared to model based estimates of expected 28-day mortality (x-axis). **SFigure 2.** Flow-diagram of the study. **SFigure 3.** Panel a: Timing of start of antimicrobial treatment in relation to onset of sepsis. Panel b: Timing of surgical source control in relation to onset of sepsis. Panel c: Cumulative proportion of patients receiving antimicrobial treatment after onset of sepsis within the first 48 hours. Panel d: Cumulative proportion of patients receiving surgical source control after onset of sepsis within the first 48 hours. **SFigure 4.** Crude mortality and predicted mortality depending on success of surgical source control. Presented are the risk (bars) and predicted risk (lines) across a range of time after onset of sepsis. Bold lines present the predicted risk with 95% confidence interval for a typical patient from a model adjusting for covariates, dotted lines present the predicted risk with 95% confidence interval from a model without adjusting for covariates. The effect of timing was tested for linearity by fractional polynomials at significance level 0.05; timing was treated as linear, since no significant deviation from linearity was found. Risk-adjusted OR with 95% CI: 0.991 [0.978, 1.005], p = 0.197; raw OR with 95% CI: 0.982 [0.971, 0.994], p = 0.003. N = 1595 cases did undergo surgical source control within 48 hours, of which 1592 cases had complete data on outcome and covariates for analysis. The risk-adjustment model involved the following covariates: age and gender, origin of infection, location of the patient at the onset of sepsis, focus of infection, microbiological confirmation of infection, study phase (trial vs. surveillance phase), and group the hospital was randomized to (intervention vs. control).

**Additional file 2.** **Supplemental Tables. STable 1.** Risk-adjustment-model for 28-day mortality excluding variables on severity of critical illness. **STable 2.** Risk-adjustment-model for 28-day mortality including variables on severity of critical illness. **STable 3.** Characteristics of participating hospitals. **STable 4.** Description of cases with timing of antimicrobial therapy before sepsis onset or after more than 48 hours. **STable 5.** Description of cases with timing of surgical source control before sepsis onset or after more than 48 hours.** STable 6.** Analysis of combined effects of delay in antimicrobial therapy and delay in surgical source control. **STable 7.** Effects of indicators of quality of anti-infective therapy on hospital length-of-stay of hospital survivors. **STable 8.** Sensitivity analysis on effects of indicators on quality of anti-infective therapy on 28-day-mortality adjusting for covariates including measures on severity of critical illness.

## Data Availability

All data requests should be submitted to Dr. Bloos (Frank.Bloos@med.uni-jena.de) for consideration. Access to the anonymised data might be granted following review by the Center for Sepsis Control and Care (CSCC) and the Jena University Hospital.

## Abbreviations

ICU: Intensive care unit
IMC: Intermediate care unit
MEDUSA trial: Medical Education for Sepsis Source Control and Antibiotics trial
OR: Odds ratio
