# Supplementary material for: Adverse effects of delayed antimicrobial treatment and surgical source control in adults with sepsis: results of a planned secondary analysis of a cluster-randomized controlled trial
Source: Crit Care. 2022 Feb 28;26:51. doi: 10.1186/s13054-022-03901-9 (PMC8883454; doi:10.1186/s13054-022-03901-9)
Supplement: Supplementary file 1 — Additional file 1. Supplemental Figures. SFigure 1. Calibration belt of the risk-adjustment-model for 28-day-mortality. The figure shows observed 28-day mortality (y-axis) compared to model based estimates of expected 28-day mortality (x-axis). SFigure 2. Flow-diagram of the study. SFigure 3. Panel a: Timing of start of antimicrobial treatment in relation to onset of sepsis. Panel b: Timing of surgical source control in relation to onset of sepsis. Panel c: Cumulative proportion of patients receiving antimicrobial treatment after onset of sepsis within the first 48 hours. Panel d: Cumulative proportion of patients receiving surgical source control after onset of sepsis within the first 48 hours. SFigure 4. Crude mortality and predicted mortality depending on success of surgical source control. Presented are the risk (bars) and predicted risk (lines) across a range of time after onset of sepsis. Bold lines present the predicted risk with 95% confidence interval for a typical patient from a model adjusting for covariates, dotted lines present the predicted risk with 95% confidence interval from a model without adjusting for covariates. The effect of timing was tested for linearity by fractional polynomials at significance level 0.05; timing was treated as linear, since no significant deviation from linearity was found. Risk-adjusted OR with 95% CI: 0.991 [0.978, 1.005], p = 0.197; raw OR with 95% CI: 0.982 [0.971, 0.994], p = 0.003. N = 1595 cases did undergo surgical source control within 48 hours, of which 1592 cases had complete data on outcome and covariates for analysis. The risk-adjustment model involved the following covariates: age and gender, origin of infection, location of the patient at the onset of sepsis, focus of infection, microbiological confirmation of infection, study phase (trial vs. surveillance phase), and group the hospital was randomized to (intervention vs. control). [file 13054_2022_3901_MOESM1_ESM.docx]

Additional File 1: Supplemental Figures

**
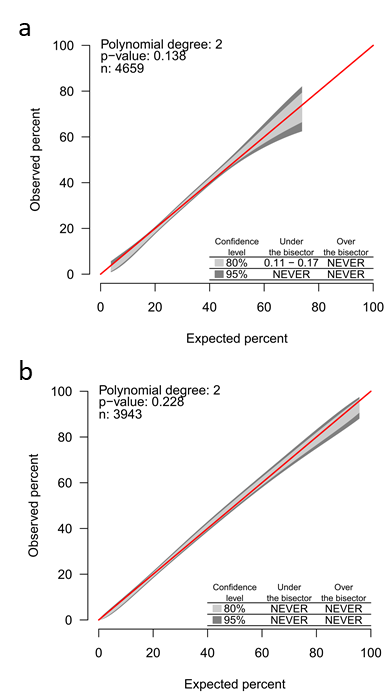
**

**SFigure 1.** Calibration belt of the risk-adjustment-model for 28-day-mortality. The figure shows observed 28-day mortality (y-axis) compared to model based estimates of expected 28-day mortality (x-axis).

Panel a: Calibration belt for the risk-model including the covariates age and gender, origin of infection, location of the patient at the onset of sepsis, focus of infection, microbiological confirmation of infection, study phase (trial vs. surveillance phase), and group the hospital was randomized to (intervention vs. control during the trial phase). Upper and lower bounds of the 95% confidence interval do not cross the red line representing equality of observed and expected mortality. This as well as the Hosmer-Lemeshow test (not significant with p = 0.138) confirm adequate calibration of the risk-adjustment model.

Panel b: Calibration belt for the risk-model used for sensitivity analysis additionally including as covariates vasopressor use at sepsis onset or within the first 12 hrs, and the maximum values within the first 24 hours after sepsis onset of SAPS-II, Lactate in mmol/l, platelets, and base excess. Upper and lower bounds of the 95% confidence interval do not cross the red line representing equality of observed and expected mortality. This as well as the Hosmer-Lemeshow test (not significant with p = 0.228) confirm adequate calibration of the risk-adjustment model.


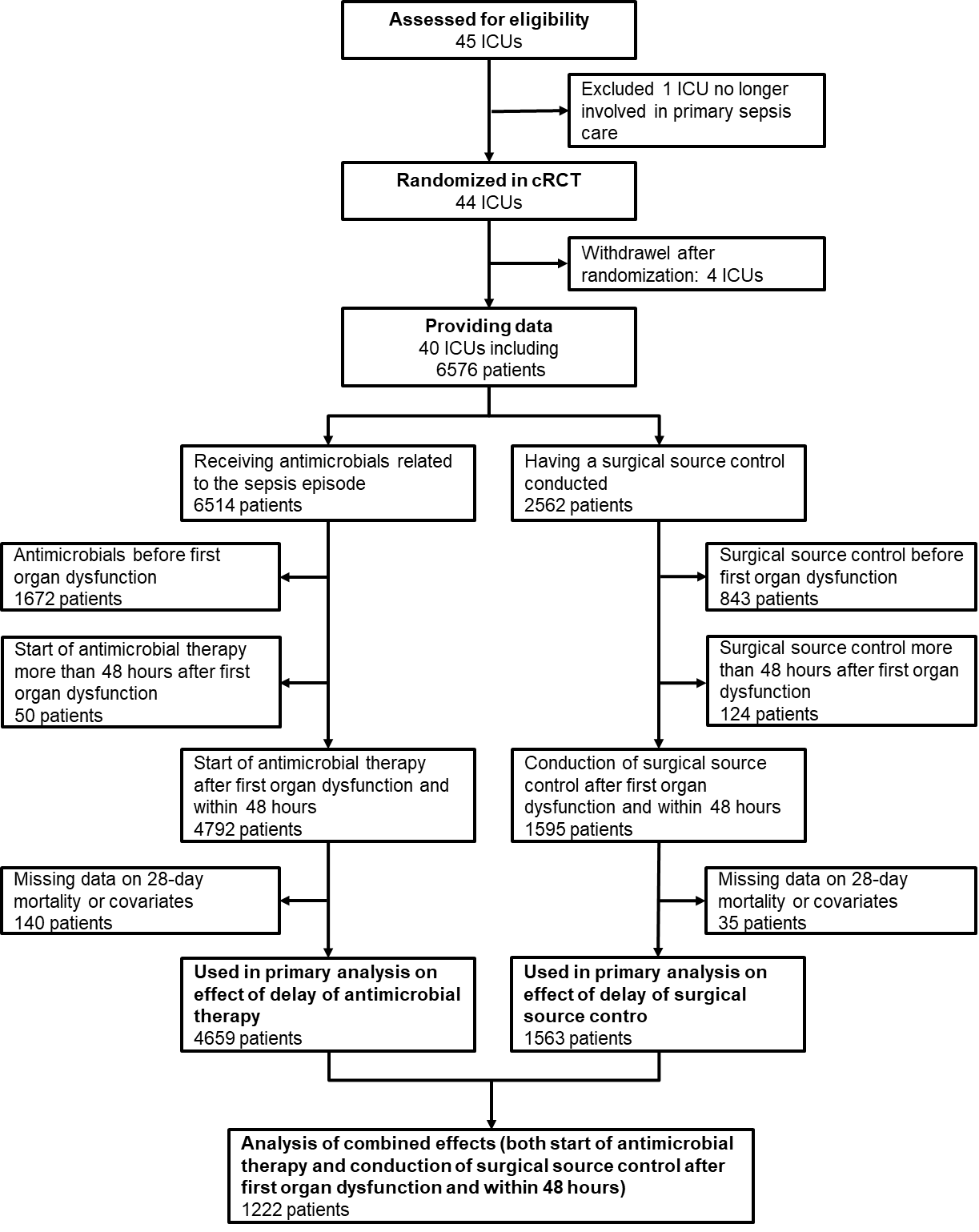


**SFigure 2.** Flow-diagram of the study

cRCT: cluster-randomized controlled trial


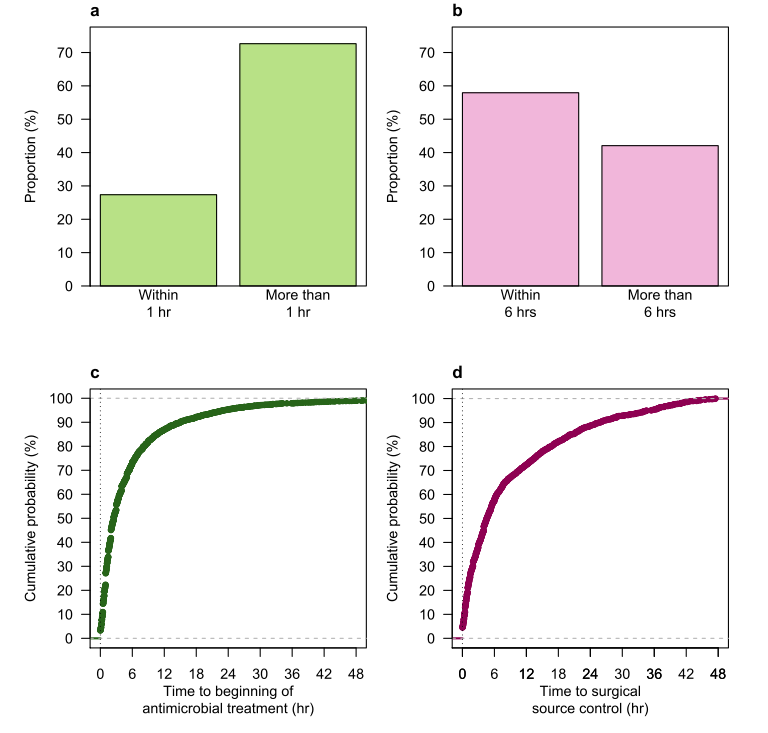


**SFigure 3.** Panel a: Timing of start of antimicrobial treatment in relation to onset of sepsis. Panel b: Timing of surgical source control in relation to onset of sepsis. Panel c: Cumulative proportion of patients receiving antimicrobial treatment after onset of sepsis within the first 48 hours. Panel d: Cumulative proportion of patients receiving surgical source control after onset of sepsis within the first 48 hours.


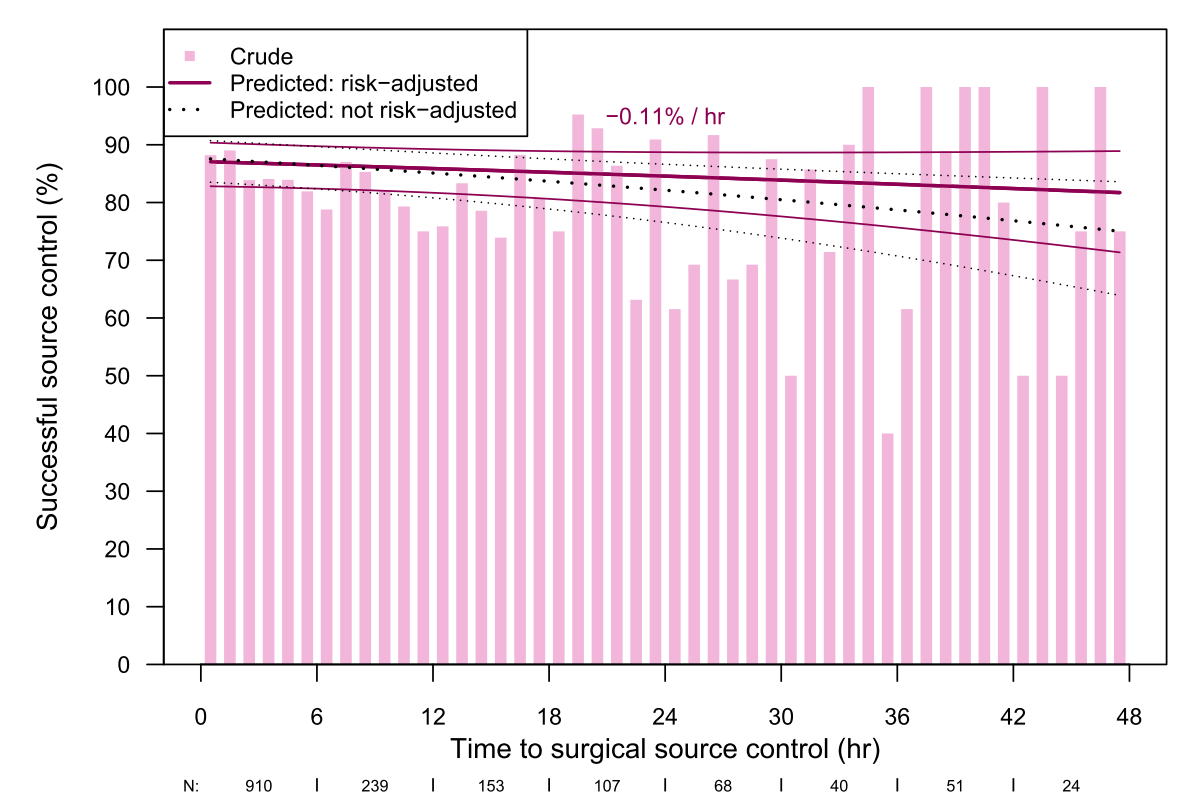


**SFigure 4.** Crude mortality and predicted mortality depending on success of surgical source control. Presented are the risk (bars) and predicted risk (lines) across a range of time after onset of sepsis. Bold lines present the predicted risk with 95% confidence interval for a typical patient from a model adjusting for covariates, dotted lines present the predicted risk with 95% confidence interval from a model without adjusting for covariates. The effect of timing was tested for linearity by fractional polynomials at significance level 0.05; timing was treated as linear, since no significant deviation from linearity was found. Risk-adjusted OR with 95% CI: 0.991 [0.978, 1.005], p = 0.197; raw OR with 95% CI: 0.982 [0.971, 0.994], p = 0.003. N = 1595 cases did undergo surgical source control within 48 hours, of which 1592 cases had complete data on outcome and covariates for analysis. The risk-adjustment model involved the following covariates: age and gender, origin of infection, location of the patient at the onset of sepsis, focus of infection, microbiological confirmation of infection, study phase (trial vs. surveillance phase), and group the hospital was randomized to (intervention vs. control).
