# Supplementary material for: Adverse effects of delayed antimicrobial treatment and surgical source control in adults with sepsis: results of a planned secondary analysis of a cluster-randomized controlled trial
Source: Crit Care. 2022 Feb 28;26:51. doi: 10.1186/s13054-022-03901-9 (PMC8883454; doi:10.1186/s13054-022-03901-9)
Supplement: Supplementary file 2 — Additional file 2. Supplemental Tables. STable 1. Risk-adjustment-model for 28-day mortality excluding variables on severity of critical illness. STable 2. Risk-adjustment-model for 28-day mortality including variables on severity of critical illness. STable 3. Characteristics of participating hospitals. STable 4. Description of cases with timing of antimicrobial therapy before sepsis onset or after more than 48 hours. STable 5. Description of cases with timing of surgical source control before sepsis onset or after more than 48 hours. STable 6. Analysis of combined effects of delay in antimicrobial therapy and delay in surgical source control. STable 7. Effects of indicators of quality of anti-infective therapy on hospital length-of-stay of hospital survivors. STable 8. Sensitivity analysis on effects of indicators on quality of anti-infective therapy on 28-day-mortality adjusting for covariates including measures on severity of critical illness. [file 13054_2022_3901_MOESM2_ESM.docx]

Additional File 2: Supplemental Tables

**STable 1. Risk-adjustment-model for 28-day mortality excluding variables on severity of critical illness**

| **Variable** | **Effect** | **Odds-ratio (95% confidence interval)** | ***P*-value of Wald-test^b^** | ***P*-value of likelihood-ratio-test^c^** |
| --- | --- | --- | --- | --- |
| Age^a^ | $\left( \frac{age}{100} \right)^{3}$ | 13.274 (8.943, 19.704) |  | ≤0.001 |
| Gender, reference: female | male | 0.95 (0.831, 1.087) |  | 0.459 |
| Origin of infection, reference: Community acquired | Nosocomial (ICU/IMC) | 0.969 (0.794, 1.182) | 0.754 | 0.54 |
|  | Nosocomial (general ward) | 1.075 (0.902, 1.281) | 0.42 |  |
| Location at onset of sepsis, reference: ICU/IMC | Ambulance service/emergency department | 0.807 (0.66, 0.987) | 0.037 | 0.06 |
|  | Operating room | 0.761 (0.591, 0.979) | 0.034 |  |
|  | General ward | 0.959 (0.777, 1.183) | 0.697 |  |
| Focus of infection: respiratory | yes | 1.279 (1.059, 1.545) |  | 0.011 |
| Focus of infection: abdominal | yes | 1.284 (1.048, 1.574) |  | 0.017 |
| Focus of infection: urogenital | yes | 0.689 (0.547, 0.868) |  | 0.001 |
| Focus of infection: bones/soft tissue/wound | yes | 1.333 (1.053, 1.687) |  | 0.018 |
| Focus of infection: other/unknown | yes | 1.457 (1.162, 1.827) |  | 0.001 |
| Infection microbiologically confirmed | yes | 1.199 (1.031, 1.395) |  | 0.018 |
| Study group and phase, ref: Group 1/Phase 1 | Group 1/Phase 2 | 0.873 (0.731, 1.042) | 0.133 | 0.287 |
|  | Group 2/Phase 1 | 0.796 (0.586, 1.081) | 0.144 |  |
|  | Group 2/Phase 2 | 0.792 (0.569, 1.102) | 0.166 |  |

N = 4792 cases received their first antimicrobial treatment between sepsis onset and 48 hours. The model was based on *N* = 4659 (97%) cases with complete data on outcome and predictors, showed a discrimination of *AUC* = 0.669 (95% CI: 0.653-0.686), and an explained variance of *R^2^*= 0.075 (95% CI: 0.061-0.09). ICU: intensive care unit. IMC: intermediate care unit.

^a^ Variable entered as a fractional polynomial

^b^ *P*-value obtained by Wald-test for comparison with the reference category

^c^ *P*-value obtained by likelihood-ratio-test as overall test of significance of a variable

.

**STable 2. Risk-adjustment-model for 28-day mortality including variables on severity of critical illness**

| **Variable** | **Effect** | **Odds-ratio (95% confidence interval)** | ***P*-value of Wald-test^b^** | ***P*-value of likelihood-ratio-test^c^** |
| --- | --- | --- | --- | --- |
| Age^a^ | $\left( \frac{age}{100} \right)$ | 22.4 (11.565, 43.386) |  | ≤0.001 |
| Gender, reference: female | male | 0.882 (0.754, 1.031) |  | 0.114 |
| Origin of infection, reference: Community acquired | Nosocomial (ICU/IMC) | 1.227 (0.971, 1.55) | 0.087 | 0.18 |
|  | Nosocomial (general ward) | 1.163 (0.946, 1.429) | 0.152 |  |
| Location at onset of sepsis, reference: ICU/IMC | Ambulance service/emergency department | 0.761 (0.601, 0.963) | 0.023 | 0.142 |
|  | Operating room | 0.856 (0.64, 1.145) | 0.295 |  |
|  | General ward | 0.924 (0.722, 1.182) | 0.529 |  |
| Focus of infection: respiratory | yes | 1.265 (1.017, 1.572) |  | 0.035 |
| Focus of infection: abdominal | yes | 0.978 (0.774, 1.236) |  | 0.854 |
| Focus of infection: urogenital | yes | 0.756 (0.58, 0.985) |  | 0.037 |
| Focus of infection: bones/soft tissue/wound | yes | 1.389 (1.06, 1.819) |  | 0.018 |
| Focus of infection: other/unknown | yes | 1.166 (0.894, 1.521) |  | 0.257 |
| Infection microbiologically confirmed | yes | 1.235 (1.035, 1.474) |  | 0.019 |
| SAPS-II^a^ | $\left( \frac{SAPSII}{100} \right)$ | 11.153 (6.501, 19.135) |  | ≤0.001 |
| Lactate mmol/l^a^ | $\frac{lactate}{10}$ | 3.714 (2.671, 5.164) | ≤0.001 | ≤0.001 |
|  | $\left( \frac{lactate}{10} \right)^{3}$ | 0.894 (0.842, 0.949) | ≤0.001 |  |
| Platelets^a^ | $\left( \frac{platelets}{100} \right)^{0.5}$ | 0.152 (0.075, 0.309) | ≤0.001 | ≤0.001 |
|  | $\left( \frac{platelets}{100} \right)^{1}$ | 1.667 (1.315, 2.114) | ≤0.001 |  |
| Base excess^a^ | $\left( \frac{base excess+30}{10} \right)^{2}$ | 0.708 (0.608, 0.824) | ≤0.001 | ≤0.001 |
|  | $\left( \frac{base excess+30}{10} \right)^{2}*\log\left( \frac{base excess+30}{10} \right)$ | 1.236 (1.129, 1.353) | ≤0.001 |  |
| Vasopressor use at sepsis onset or within the first 12 hrs | yes | 1.086 (0.89, 1.324) |  | 0.417 |
| Study group and phase, reference: Group 1/Phase 1 | Group 1/Phase 2 | 0.971 (0.795, 1.185) | 0.77 | 0.598 |
|  | Group 2/Phase 1 | 0.811 (0.601, 1.095) | 0.171 |  |
|  | Group 2/Phase 2 | 0.83 (0.597, 1.152) | 0.265 |  |

N = 4792 cases received their first antimicrobial treatment between sepsis onset and 48 hours. The model was based on *N* = 3943 (82%) cases with complete data on outcome and predictors, showed a discrimination of *AUC* = 0.759 (95% CI: 0.743-0.775), and an explained variance of *R^2^*= 0.181 (95% CI: 0.159-0.203). ICU: intensive care unit. IMC: intermediate care unit.

^a^ Variable entered as a fractional polynomial

^b^ *P*-value obtained by Wald-test for comparison with the reference category or test of a part of a fractional polynomial

^c^ *P*-value obtained by likelihood-ratio-test as overall test of significance of a variable

**STable 3. Characteristics of participating hospitals**

| **Variable** | **Participating hospitals (N = 40)** |
| --- | --- |
| Teaching status: Non-teaching | 6 (15) |
| Teaching | 25 (62.5) |
| University | 9 (22.5) |
| Hospital operator: non-profit | 5 (12.5) |
| public | 23 (57.5) |
| private | 12 (30) |
| Level of care: Primary | 12 (30) |
| Secondary | 12 (30) |
| Tertiary | 16 (40) |
| Number of hospital beds | 596 [418, 1031.75] |
| Number of ICU beds | 17 [10, 31] |
| ICU cases per year | 1225 [993.75, 2125] |
| Inhospital microbiology department | 28 (70) |

Descriptive statistics given as N (%) or median [interquartile range].

**STable 4: Description of cases with timing of antimicrobial therapy before sepsis onset or after more than 48 hours**

|  |  |  | Timing of beginning of antimicrobial therapy | | | |
| --- | --- | --- | --- | --- | --- | --- |
| Variable | No. of patients with complete data | All patients (N = 6514) | Before sepsis onset (N = 1672) | Between sepsis onset and 48 hours (N = 4792) | After more than 48 hours (N = 50) | P-value |
| Age | 6513 | 70 [59, 77] | 69 [58, 76]^a^ | 70 [59, 77]^a^ | 73 [64.75, 77] | 0.003 |
| Gender: male | 6514 | 4060 (62.3%) | 1044 (62.4) | 2986 (62.3) | 30 (60) | 0.939 |
| Origin of infection: Community acquired | 6513 | 2956 (45.4%) | 663 (39.7)^a^ | 2273 (47.4)^a^ | 20 (40) | ≤0.001 |
| Nosocomial (ICU/IMC) |  | 1470 (22.6%) | 343 (20.5) | 1112 (23.2) | 15 (30) |  |
| Nosocomial (general ward) |  | 2087 (32%) | 666 (39.8) | 1406 (29.3) | 15 (30) |  |
| Location at onset of sepsis: ICU | 6514 | 3295 (50.6%) | 1040 (62.2)^a^ | 2233 (46.6)^a^ | 22 (44)^a^ | ≤0.001 |
| Emergency department |  | 1036 (15.9%) | 84 (5) | 948 (19.8) | 4 (8) |  |
| Operating room |  | 686 (10.5%) | 255 (15.3) | 428 (8.9) | 3 (6) |  |
| General ward |  | 895 (13.7%) | 191 (11.4) | 692 (14.4) | 12 (24) |  |
| Ambulance service |  | 217 (3.3%) | 5 (0.3) | 211 (4.4) | 1 (2) |  |
| IMC |  | 385 (5.9%) | 97 (5.8) | 280 (5.8) | 8 (16) |  |
| Focus of infection: respiratory | 6511 | 2664 (40.9%) | 583 (34.9)^a^ | 2057 (43)^a^ | 24 (48) | ≤0.001 |
| Focus of infection: abdominal | 6511 | 2413 (37.1%) | 741 (44.3)^a^ | 1657 (34.6)^a^ | 15 (30) | ≤0.001 |
| Focus of infection: urogenital | 6511 | 868 (13.3%) | 167 (10)^a^ | 695 (14.5)^a^ | 6 (12) | ≤0.001 |
| Focus of infection: bones/soft tissue/wound | 6511 | 716 (11%) | 179 (10.7) | 531 (11.1) | 6 (12) | 0.888 |
| Focus of infection: other/unknown | 6511 | 869 (13.3%) | 217 (13) | 644 (13.4) | 8 (16) | 0.762 |
| Infection microbiologically confirmed | 6500 | 4723 (72.7%) | 1168 (69.9)^a,b^ | 3514 (73.5)^a^ | 41 (83.7)^b^ | 0.004 |
| Bacteremia: Gram positive | 6463 | 1057 (16.4%) | 242 (14.6)^a^ | 806 (17)^a^ | 9 (18.8)^a^ | ≤0.001 |
| Gram negative |  | 878 (13.6%) | 165 (9.9) | 712 (15) | 1 (2.1) |  |
| Other/several |  | 224 (3.5%) | 54 (3.3) | 166 (3.5) | 4 (8.3) |  |
| No pathogen detected |  | 3120 (48.3%) | 823 (49.5) | 2281 (48) | 16 (33.3) |  |
| No blood culture taken |  | 1184 (18.3%) | 377 (22.7) | 789 (16.6) | 18 (37.5) |  |
| SAPS-II | 5774 | 48 [38, 60] | 45 [36, 56]^a,b^ | 49 [39, 61]^a^ | 49 [42.5, 60]^b^ | ≤0.001 |
| Lactate (mmol/l) | 6293 | 2.6 [1.6, 4.8] | 2.3 [1.5, 4.1]^a^ | 2.7 [1.6, 5]^a^ | 2.7 [1.6, 4.5] | ≤0.001 |
| Platelets | 6480 | 191 [120, 288] | 199 [127, 301]^a^ | 189 [117, 285]^a^ | 188 [120.25, 267.25] | 0.005 |
| Base excess | 6307 | -3.5 [-7.8, 2.3] | -2.8 [-6.7, 2.5]^a^ | -3.8 [-8.1, 2.2]^a,b^ | -2.4 [-5.3, 3.9]^b^ | ≤0.001 |
| Vasopressor use at sepsis onset or within the first 12 hrs | 6502 | 4890 (75.2%) | 1263 (75.6) | 3595 (75.2) | 32 (64) | 0.174 |
| 28-day mortality | 6345 | 2003 (31.6%) | 505 (31) | 1476 (31.6) | 22 (44) | 0.149 |
| Hospital length-of-stay of hospital survivors (days) | 4065 | 33 [20, 51] | 36 [22, 53]^a^ | 32 [19, 50]^a^ | 46.5 [37, 77.75]^a^ | ≤0.001 |

Descriptive statistics presented as Median [1^st^ quartile, 3^rd^ quartile] or N (%). P-values obtained by Chi-square test or Kurskal-Wallis test as appropriate. ICU: intensive care unit. IMC: intermediate care unit.

^a,b^ For each variable, columns sharing the same superscript are pairwise statistically different from each other with *p* ≤ 0.05.

**STable 5: Description of cases with timing of surgical source control before sepsis onset or after more than 48 hours**

|  |  |  | Timing of surgical source control | | | |
| --- | --- | --- | --- | --- | --- | --- |
| Variable | No. of patients with complete data | All patients (N = 2562) | Before sepsis onset (N = 843) | Between sepsis onset and 48 hours (N = 1595) | After more than 48 hours (N = 124) | P-value |
| Age | 2562 | 70 [59, 77] | 70 [60, 77] | 70 [59, 77] | 70 [59, 76.25] | 0.888 |
| Gender: male | 2562 | 1501 (58.6%) | 490 (58.1) | 934 (58.6) | 77 (62.1) | 0.703 |
| Origin of infection: Community acquired | 2562 | 1238 (48.3%) | 385 (45.7)^a^ | 795 (49.8)^a^ | 58 (46.8)^a^ | ≤0.001 |
| Nosocomial (ICU/IMC) |  | 413 (16.1%) | 84 (10) | 292 (18.3) | 37 (29.8) |  |
| Nosocomial (general ward) |  | 911 (35.6%) | 374 (44.4) | 508 (31.8) | 29 (23.4) |  |
| Location at onset of sepsis: ICU | 2562 | 1139 (44.5%) | 506 (60)^a^ | 573 (35.9)^a^ | 60 (48.4)^a^ | ≤0.001 |
| Emergency department |  | 329 (12.8%) | 17 (2) | 298 (18.7) | 14 (11.3) |  |
| Operating room |  | 613 (23.9%) | 267 (31.7) | 344 (21.6) | 2 (1.6) |  |
| General ward |  | 278 (10.9%) | 23 (2.7) | 232 (14.5) | 23 (18.5) |  |
| Ambulance service |  | 46 (1.8%) | 3 (0.4) | 39 (2.4) | 4 (3.2) |  |
| IMC |  | 157 (6.1%) | 27 (3.2) | 109 (6.8) | 21 (16.9) |  |
| Focus of infection: respiratory | 2562 | 269 (10.5%) | 75 (8.9)^a^ | 171 (10.7)^b^ | 23 (18.5)^a,b^ | 0.004 |
| Focus of infection: abdominal | 2562 | 1804 (70.4%) | 583 (69.2)^a^ | 1161 (72.8)^b^ | 60 (48.4)^a,b^ | ≤0.001 |
| Focus of infection: urogenital | 2562 | 199 (7.8%) | 71 (8.4) | 121 (7.6) | 7 (5.6) | 0.507 |
| Focus of infection: bones/soft tissue/wound | 2562 | 440 (17.2%) | 126 (14.9)^a^ | 272 (17.1)^b^ | 42 (33.9)^a,b^ | ≤0.001 |
| Focus of infection: other/unknown | 2562 | 236 (9.2%) | 93 (11)^a^ | 117 (7.3)^a^ | 26 (21)^a^ | ≤0.001 |
| Infection microbiologically confirmed | 2558 | 1958 (76.5%) | 614 (72.8)^a,b^ | 1239 (77.8)^a^ | 105 (85.4)^b^ | 0.001 |
| Bacteremia: Gram positive | 2548 | 356 (14%) | 92 (11)^a^ | 224 (14.1)^a^ | 40 (32.8)^a^ | ≤0.001 |
| Gram negative |  | 264 (10.4%) | 48 (5.7) | 206 (13) | 10 (8.2) |  |
| Other/several |  | 73 (2.9%) | 18 (2.1) | 54 (3.4) | 1 (0.8) |  |
| No pathogen detected |  | 1182 (46.4%) | 420 (50.1) | 720 (45.4) | 42 (34.4) |  |
| No blood culture taken |  | 673 (26.4%) | 261 (31.1) | 383 (24.1) | 29 (23.8) |  |
| SAPS-II | 2314 | 47 [37, 59] | 44 [35, 54]^a^ | 49 [39, 62]^a^ | 48 [36.5, 57] | ≤0.001 |
| Lactate (mmol/l) | 2491 | 3 [1.7, 5.5] | 2.4 [1.6, 4.2]^a^ | 3.4 [1.8, 6.2]^a,b^ | 2.5 [1.6, 4.2]^b^ | ≤0.001 |
| Platelets | 2549 | 207 [132, 309] | 223 [149, 320.75]^a^ | 201 [128, 306]^a^ | 176.5 [101, 265]^a^ | ≤0.001 |
| Base excess | 2502 | -4.1 [-8.38, 1.2] | -3.2 [-7, 1.7]^a^ | -5 [-9.2, 0]^a^ | -1.15 [-6.27, 4.3]^a^ | ≤0.001 |
| Vasopressor use at sepsis onset or within the first 12 hrs | 2555 | 2120 (83%) | 696 (82.8)^a^ | 1346 (84.7)^b^ | 78 (62.9)^a,b^ | ≤0.001 |
| 28-day mortality | 2505 | 783 (31.3%) | 224 (27.3)^a,b^ | 510 (32.6)^a^ | 49 (40.5)^b^ | 0.002 |
| Hospital length-of-stay of hospital survivors (days) | 1585 | 38 [23, 57] | 37 [23, 54]^a^ | 39 [23, 58]^b^ | 46.5 [32, 65.75]^a,b^ | 0.06 |

Descriptive statistics presented as Median [1^st^ quartile, 3^rd^ quartile] or N (%). P-values obtained by Chi-square test or Kurskal-Wallis test as appropriate. ICU: intensive care unit. IMC: intermediate care unit.

^a,b^ For each variable, columns sharing the same superscript are pairwise statistically different from each other with *p* ≤ 0.05.

**STable 6: Analysis of combined effects of delay in antimicrobial therapy and delay in surgical source control**

|  | **Odds ratio** | **Lower 95% CI** | **Upper 95% CI** | **P-Value** |
| --- | --- | --- | --- | --- |
| Time to antimicrobial therapy | 1.025 | 0.991 | 1.06 | 0.152 |
| Time to surgical source control | 1.011 | 0.995 | 1.027 | 0.177 |
| Interaction between both^a^ | 1 | 0.998 | 1.001 | 0.577 |

Results of generalized hierarchical model with logit link and a random intercept and adjustment for confounders. Confounders are listed in STable 1. N = 1250 cases had both timing of beginning of antimicrobial therapy and timing of surgical source control between sepsis onset and 48 hours, of which 1222 (98%) had complete data on outcome and confounders for analysis.

^a^ Interaction tests for a multiplicative effect of delay of antimicrobial therapy and delay of surgical source control. A positive interaction effect (OR > 1) would mean that a delay in both treatments would cause a more than additive increase in risk of death.

**STable 7. Effects of indicators of quality of anti-infective therapy on hospital length-of-stay of hospital survivors**

| **Variable** | **No. of cases with complete data/No. of cases** | **Effect** | **Ratio of geometric means (95% CI)** | ***P*-value of Wald-test^b^** | ***P*-value of likelihood-ratio-test^c^** |
| --- | --- | --- | --- | --- | --- |
| log(Time to antimicrobial therapy)^a^ | 2980/2988 |  | 1.02 (1, 1.03) | 0.023 |  |
| Antimicrobial therapy | 2980/2988 | 0-1 hr | 1 |  | 0.093 |
|  |  | 1-3 hrs | 1.05 (0.99, 1.11) | 0.116 |  |
|  |  | 3-6 hrs | 1.07 (1, 1.15) | 0.042 |  |
|  |  | >6 hrs | 1.08 (1.01, 1.15) | 0.025 |  |
| log(Time to source control)^a^ | 956/959 |  | 1.04 (1.01, 1.07) | 0.005 |  |
| Surgical source control | 956/959 | 0-1 hr | 1 |  | 0.019 |
|  |  | 1-3 hrs | 1.01 (0.88, 1.17) | 0.863 |  |
|  |  | 3-6 hrs | 1.09 (0.93, 1.27) | 0.273 |  |
|  |  | >6 hrs | 1.2 (1.04, 1.38) | 0.013 |  |
| Success of source control | 956/959 | no | 1 |  |  |
|  |  | yes | 0.72 (0.61, 0.86) | ≤0.001 |  |

Results of hierarchical linear models with a random intercept and logarithmic transformation of the outcome variable and adjustment for covariates. Confounders are listed in STable 1. Analysis conducted among hospital survivors.

^a^ Times were log-transformed because of deviations from normal distribution.

^b^ *P*-value obtained by Wald-test for comparison with the reference category

^c^ *P*-value obtained by likelihood-ratio-test as overall test of significance of a variable

**STable 8. Sensitivity analysis on effects of indicators on quality of anti-infective therapy on 28-day-mortality adjusting for covariates including measures on severity of critical illness**

| **Variable** | **No. of cases with complete data/No. of cases** | **Effect** | **Oddds-ratio (95% CI)** | ***P*-value of Wald-test^a^** | ***P*-value of likelihood-ratio-test^b^** |
| --- | --- | --- | --- | --- | --- |
| Time to antimicrobial therapy | 3943/4792 | effect per hour | 1.03 (1.02, 1.04) | ≤0.001 |  |
| Antimicrobial therapy | 3943/4792 | 0-1 hr | 1 |  | ≤0.001 |
|  |  | 1-3 hrs | 1.16 (0.94, 1.42) | 0.171 |  |
|  |  | 3-6 hrs | 1 (0.79, 1.26) | 0.973 |  |
|  |  | >6 hrs | 1.53 (1.23, 1.9) | ≤0.001 |  |
| Time to surgical source control | 1343/1595 | effect per hour | 1.02 (1.01, 1.04) | 0.001 |  |
| Surgical source control | 1343/1595 | 0-1 hr | 1 |  | 0.057 |
|  |  | 1-3 hrs | 1.11 (0.68, 1.82) | 0.677 |  |
|  |  | 3-6 hrs | 1.12 (0.66, 1.91) | 0.666 |  |
|  |  | >6 hrs | 1.64 (1.01, 2.67) | 0.044 |  |
| Success of surgical source control | 1343/1595 | no | 1 |  |  |
|  |  | yes | 0.15 (0.1, 0.22) | ≤0.001 |  |

Results of logistic hierarchical linear models with a random intercept adjusting for confounders including measures on severity of critical illness during the first 24 hours after onset of sepsis. Confounders are listed in STable 2.

^a^ *P*-value obtained by Wald-test for comparison with the reference category

^b^ *P*-value obtained by likelihood-ratio-test as overall test of significance of a variable
